# Indications and recommended approach for surgical intervention of metastatic disease to the gallbladder

**DOI:** 10.1186/1477-7819-8-80

**Published:** 2010-09-10

**Authors:** Zarrish S Khan, James Huth, Payal Kapur, Sergio Huerta

**Affiliations:** 1 Department of Surgery, UT Southwestern Medical Center, Harry Hines Blvd, Dallas 75219, USA; 2 Department of Pathology, UT Southwestern Medical Center, Harry Hines Blvd, Dallas 75219, USA

## Abstract

Metastatic disease to the gallbladder is unusual. The most common malignancy metastatic to the gallbladder is melanoma, followed by renal cell carcinoma (RCC) and breast cancer. Due to the unusual nature of the disease, there are no trials available for review. Thus, the management for these patients has been based on institutional experience and review of case series. The indications for surgical intervention for melanoma are metastatic disease discrete to the gallbladder and biliary symptoms, which are uncommon for melanoma, but might occur due to cystic duct obstruction culminating in cholecystitis. Laparoscopic cholecystectomy without a lymphadenectomy is emerging as the preferred approach for this metastatic deposit. The vast majority of patients with metastases to the gallbladder from RCC carry a good prognosis and a laparoscopic cholecystectomy should be considered. Patients with metastases to the gallbladder from the breast classically present with biliary symptoms and commonly undergo a laparoscopic cholecystectomy, which invariably demonstrates a deposit in the gallbladder from lobular breast cancer. In the present report, we review the indications for surgical intervention from various malignancies metastatic to the gallbladder and the current consensus for the laparoscopic approach from the diverse metastatic deposits to the gallbladder.

## Metastasis to the Gallbladder

An autopsy analysis of 1,000 consecutive cases of malignancies revealed an incidence of metastasis to the gallbladder of 5.8% [1]. By comparison, the incidence of metastasis to the most common organs was 49.5%, 49.4%, and 46.5% for abdominal lymph nodes, liver and lungs respectively. Thus, metastatic disease to the gallbladder is relatively rare.

In a Korean report, 20 cases of metastasis to the gallbladder were discussed [2]. The most common source of metastasis was direct invasion from intra-abdominal cancers including colon and gastric malignancies. However, the country of origin of this report, where gastric cancer has high prevalence, limits any generalizations from this series.

In our review of the literature, because the typical course of metastasis to the gallbladder is via hematogenous spread [3], the most commonly metastatic disease to the gallbladder was from melanoma followed by renal cell carcinoma and then breast cancer. Other cancers that have been reported, we have grouped in the miscellaneous category.

In the present review, we discuss whether surgical intervention has the same recommendations for a metastatic deposit from melanoma compared to breast cancer. We also interrogate the role of laparoscopic cholecystectomy in such approach. A discussion of a case in our own experience is a pertinent good start.

## Case Report

**Figure 1:**
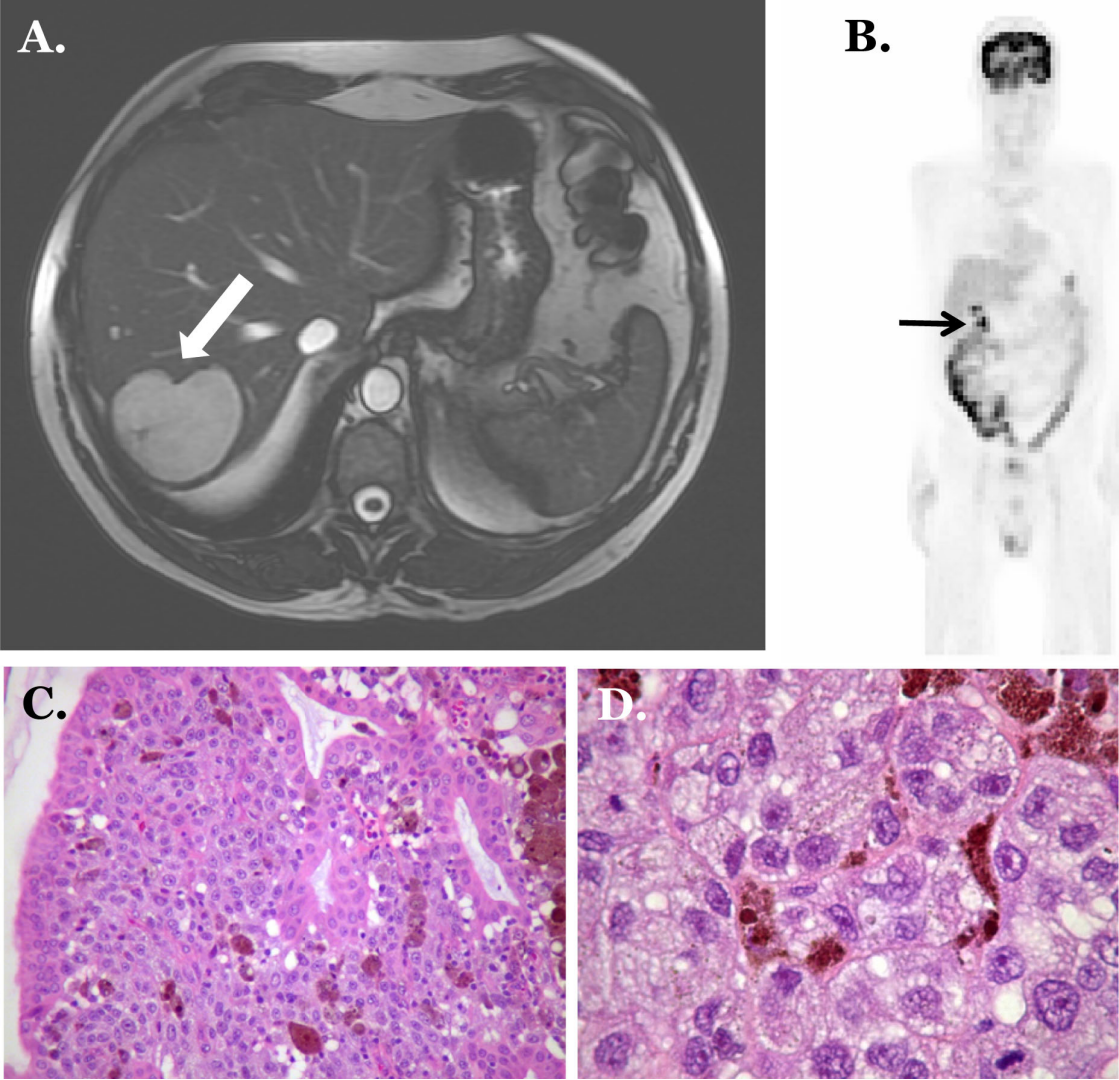
**MRI image of metastatic melanoma in liver**. At this presentation the patient did not have evidence of gallbladder disease **(A)**. PET image after high dose interleukin and chemotherapy shows decreased uptake in liver lesion however a new area of activity is now evident in the region of gallbladder **(B)**. Photomicrograph of metastatic malignant melanoma to gall bladder mucosa showing large cells with round to oval nuclei, prominent nucleoli, and intracytoplasmic pigment: Hematoxylin and Eosin stain, × 100 magnification **(C)**. Hematoxylin and Eosin stain, × 400 magnification **(D)**.

A 53 year-old man referred to the surgical oncology clinic after an episode of abdominal pain that revealed an isolated right liver lobe mass (Figure 1), which subsequently demonstrated melanoma on biopsy. Sixteen years previously, he had undergone resection of a facial melanoma. In view of the patient's excellent performance status, long latency from primary lesion and limited metastatic disease, he underwent aggressive loco-regional treatment. A metastasectomy was attempted for liver lesion. However, intra-operatively the tumor burden was substantial such that a safe operation for cure could not be undertaken. Additionally, in subsequent studies he had lung and brain metastases, for which he received systemic therapy including high dose interleukin-2 and chemotherapy consisting of cisplatin, dacarbizine, vinblastine followed by temozolomide.

He had a good response to these modalities to the point of complete regression of the liver and lung lesions as assessed by Positron Emission Tomography (PET) scan. During the same examination, a new gallbladder lesion that had high uptake was identified (Figure 1; panel B). He underwent open cholecystectomy. Gross exam revealed an obvious dark-pigmented lesion 4.8 × 2.6 × 2.2 cm in dimensions. No gallstones were identified. Microscopic examination from this lesion confirmed metastatic melanoma to gallbladder mucosa with all margins free of tumor (Figure 1; panels C & D). An additional 5.8 × 3.7 × 2.5 cm peri-portal focus of melanoma was also identified and resected. The patient made an uneventful post-operative recovery. However, he subsequently progressed with widely metastatic disease in the central nervous system and died four months after surgical intervention for the gallbladder.

## Discussion and review of the literature

### Melanoma

The aggressive nature of melanoma and the potential to metastasize to any organ in the body is demonstrated in a review of the literature reported by Dong [3], which contained the highest cases of metastasis to the gall bladder originating from melanoma. While cutaneous melanoma is known to metastasize to any organ, metastatic disease from this malignancy to the GI tract occurs with a frequency of 2%-4% [4]. Of these, the gallbladder is a site of metastasis with a frequency of 15% [4]. Of all metastatic lesions to the gallbladder, melanoma accounts for 30-60% of these cases [5].

While primary melanoma to the gallbladder is possible due to the presence of melanocyte migration during development, the need to differentiate primary and metastatic melanoma to the gallbladder (MMGB), is the center of current debate [6,7] and is beyond the scope of this review [8]. However, primary melanoma of the GB might present more frequently with symptoms and because it is limited to the GB, the prognosis might be superior compared to MMGB [3].

In the majority of cases, an asymptomatic presentation is the rule for MMGB. However, when symptoms occur, it is the result of cystic duct obstruction leading to cholecystitis [3]. The vast majority of patients with metastases to the gallbladder have evidence of disseminated disease and only a small percentage demonstrate metastatic deposits exclusively in the gallbladder [3]. The median survival of patients with metastatic disease to the gallbladder is 8.5 months [9].

The largest case series available today originates from the Duke University Medical Center, which documented 19 cases of MMGB in 1999 [3]. The main finding from this series was that none of eleven patients were alive one year after the diagnosis if they had disease outside of the gallbladder. However, if the metastatic deposit was limited to the GB, all of six patients were alive one year after the diagnosis. They reported the longest living subject with MMGB cancer who was alive 13.8 years at the time of the report [3]. The authors recommended surgical intervention for localized disease.

### Indications for surgical intervention for melanoma metastatic to the gallbladder

While survival is important in the management of patients with metastatic disease to the gallbladder, the indications for surgical intervention in this group of patients remain unclear. A group from the Sloan Kettering Cancer Center reported their experience with 13 cases of metastatic melanoma to the gallbladder in 2007 [10]. The goal of this analysis was to identify factors that might dictate surgical treatment. Univariate analysis showed that patients who had biliary symptoms and patients with metastatic deposits exclusively in the gallbladder had an increased survival. Additionally, patients who underwent a cholecystectomy had a 10 month increase in survival compared to those who did not. Nine patients had a cholecystectomy and two cases of port-metastases occurred [10]. The authors concluded that with proper patient selection, palliative surgery is an option. Because of the two cases of port-metastases and the small sample size of their study, the authors were unable to advice whether a cholecystectomy via a laparoscopy was a viable alternative.

In a report of a 58 year-old man with MMGB who presented with acute cholecystitis, the authors' review of the literature indicated that while symptoms are uncommon, when present, acute cholecystitis is most likely clinical presentation, followed by biliary obstruction leading to cholangitis. Fistula formation and hematobilia might also occur [11], similar to conclusions in previous reports [12]. This patient was treated via an open cholecystectomy because of a prior operation. The authors concluded that while the treatment of metastatic deposits is unclear, the aim should be palliation, reduction of complications and survival improvement [11].

In one case, a 63 year-old woman underwent an oncologic operation for gallbladder cancer and during pathological examination she had metastatic melanoma to the gallbladder [13]. This patient had a melanoma resected from her back prior to presentation and two years subsequent to the oncologic operation for a misdiagnosed gallbladder malignancy, she developed tonsilar and pulmonary metastatic disease. The authors suggested that a known diagnosis of metastasis prior to surgical intervention would have avoided extensive hepatic and nodal dissection [3].

In a separate report, twenty-one months following resection of a melanoma of an upper arm, a 30 year-old woman develop MMGB [14]. This patient was treated via an open cholecystectomy. The authors concluded that surgical intervention was the only modality of choice that offered an effective treatment [14].

A 32 year-old woman had a melanoma excised from her shoulder and a year later she began having symptoms of biliary colic. She underwent an open cholecystectomy and pathological examination demonstrated metastatic melanoma. She later developed brain metastases and died from wide spread disease four months following this event [15]. In their review, the authors indicated that the most common presentation for a patient with MM was cholecystitis without cholelithiasis [15]. Their analysis showed that most cases of MM disease to the gallbladder benefited from a palliative cholecystectomy. Thus, surgical management of MMGB has been advocated even in the presence of disseminated disease for palliative purposes [16].

### The role of laparoscopic cholecystectomy in the management of MMGB

Laparoscopic cholecystectomy (LC) has been described for the management of these lesions. The first report of LC for MMGB was described by Velez during an incidental finding in 1995 [9]. Two years later, Seeling successfully treated a patient with known MMGB [4]. Since then, sporadic reports have emerged in the literature: two by Kholer [17], three by Katz [10], one by Tuveri [18], one by Gould [19], and one by Marone [20]. The last of these, included a review of all the laparoscopic cases and concluded that more data were needed prior to proceeding with recommendation for or against LC for MMGB [20]. These data are discussed below.

In one case, a 54 year-old man with MMGB was treated by LC. The authors concluded that LC was a feasible approach when the disease was limited to the gallbladder, but indicated that an exploratory laparotomy might detect other sites of metastasis by palpable inspection not captured by pre-operative imaging modalities [20]. The question as to what to do if such disease is identified while subjecting a patient to the high risk of a laparotomy still remains.

In another case, a 48 year-old man with a melanoma of his flank and axillary metastasis, developed colicky pain, two years after the resection. Diagnostic tests revealed a polypoid lesion and a LC was undertaken. Examination of the GB revealed MM. In their review, the authors indicated that only 60 cases of MM had been reported in the world literature and 81% of patients had symptoms leading to a cholecystectomy [21]. Their analysis showed that while there is a concern for port-site involvement with a LC, this incidence might be similar in open cases [22]. The authors concluded that while more studies were needed, LC is the strategy of choice for patients with localized disease [21].

In a report of a 37 year-old woman with metastatic melanoma to the gallbladder who presented with acute cholecystitis, the authors treated this patient via a LC and lymphadenectomy of the hepatoduodenal ligament [18]. In their review, the authors divided the management of melanoma of the gallbladder to primary and metastatic lesions. Their review of the literature indicated that the most optimal management of primary melanoma was via an open cholecystectomy with lymphadenectomy and possible liver resection. In cases of metastatic melanoma, an open cholecystectomy was adequate. They suggested that role of a LC for the management of MMGB was still in its infancy to be able to draw meaningful conclusions. There was recognition of a possible disruption of the GB leading to port site metastasis and peritoneal disease. However, meticulous dissection and employment of an endobag should minimize these complications. The nature of the metastatic deposit growing intraluminally and the fact that lymphadenecetomy is not required for metastatic disease; the need for additional interventions is negated [18]. Similarly, a 65 year-old and a 49 year-old patient with known MMGB were treated via LC without compilations. The authors emphasized the intraluminal growth of the lesions negating the need for a hepatoduodenal ligament lymphadenopathy [17].

The short spectrum of clinical presentation was documented in two cases of MMGB [23]. In one case, a 52 year-old had biliary colic and MM melanoma was discovered after a LC. In a second case, a 60 year-old underwent a LC for known MMGB. While both of these cases were treated via LC and no port metastases of peritoneal disease occurred similar to other reports [4,17], the authors recognized the limitation of the available evidence to be able to recommend this strategy uniformly for all cases of MMGB [23].

In a case of MMGB and metastatic melanoma to the small bowel with an unknown primary, a 58 year-old man presented with abdominal pain and vomiting culminating in a small bowel obstruction [12]. This patient underwent a laparotomy with a cholecystectomy and five small bowel resections for the management of these lesions. The authors concluded that the role of LC for MMGB was not clear [12]. Another patient who was a 75 year-old woman with recurrent melanoma had multiple lesions of the gallbladder that represented a spectrum of the malignant melanoma within the gallbladder. The patient was treated via a LC [19].

Thus, for MMGB the clear indications for surgical intervention are disease limited to the gallbladder and biliary symptoms. Other indications need to be considered in a case-to-case basis. While, there are not sufficient cases to comment on the feasibility of performing these cases laparoscopically, several reports have undertaken this approach and with the increasing role of laparoscopic surgery, it is likely that most of these cases are going to be attempted via a laparoscopic approach.

### Renal Cell Carcinoma (RCC)

The second most common malignancy to metastasize to the gallbladder is renal cell carcinoma (RCC). The largest reported series was documented in 2006 and consisted of 24 non- consecutive cases. In this series, the average age of the patients was 64.5 ± 2.4 year-old and 87.5% were men. 58.3% of the lesions were metachronous deposits presenting with an average of 9.1 ± 1.8 years after the primary diagnosis of RCC. Fifty percent of patients were alive and 37.5% had no evidence of disease at the time of the report with the longest follow up of 6 years [24]. In this series, 58.3% of patients were treated via an open cholecystectomy, 29.1 by an extended cholecystectomy and 12.5% via a LC [24].

A second large series was reported in 2008 and included 13 cases [25]. In this review, the average age of patients was 60.0 ± 3.4 year-old, 69% of patients had metachronous lesions with an average time to presentation of 5.4 ± 2.7 years, 69.2% of patients were alive at an average follow up of 26.0 ± 6.4 months. All the patients in this series underwent a cholecystectomy with or without a radical nephrectomy. Seventy-percent of patients were treated exclusively via a LC.

Since this series, several case reports have been documented. In one case a 64 year-old woman presented with biliary colic and underwent a successful LC. Pathological examination revealed a metachronous RCC lesion with a median interval of seven years [26]. This manuscript indicated 23 cases of RCC metastatic to the gallbladder had been reported and that 39% of these patients remained free of disease at a longest follow up of 6 years.

An earlier and smaller series from Korea reported eight cases of metachronous lesions to the gallbladder from RCC. In this series, the median age was 60.6 ± 5.5, only two patients underwent a laparoscopic cholecystectomy, two were treated via an open cholecystectomy and the rest via a laparotomy [27].

The body of evidence from these series and case reports of RCC metastatic to the gallbladder indicate that the metastatic lesions are typically metachronous. Patient with these lesions carry a good prognosis and a LC is an adequate form of treatment.

### Metastatic Breast Cancer to the Gallbladder (MBGB)

Even in comparison with the rarity of metastatic lesions to the gallbladder from melanoma and renal cell cancer, breast cancer metastatic to the gallbladder is even more unusual. Autopsy studies indicate that the gallbladder is affected with a frequency of 4-7% [28]. Only a few cases of MBGB appear in literature and are reviewed in Table 1.

**Table 1:** Summary of case reports of breast cancer metastatic to gallbladder.

Author (year)	Age	Symptoms	Histology	Outcome
Beaver (1986) [34]	73	Cholecystitis	Lobular	NM
Rubin (1989) [43]	55	Biliary colic	Lobular	NM
Pappo (1991) [44]	NM	Obstructive jaundice	Lobular	Alive (16 months)
Crawford (1996) [35]	66	Cholecystitis	Ductal	Alive -1 year
Crawford (1996) [35]	57	Cholecystitis	Lobular	Died-3 years
Shah (2000) [38]	78	Bile peritonitis-necrotic gallbladder perforation	NM (description of Lobular)	Died-5 days
Boari (2005) [31]	81	Cholecystitis	Undifferentiated	Not mentioned
Doval (2006) [33]	NM	Cholecystitis	Lobular (signet)	Died 'few months'
Murguia (2006) [32]	62	Biliary	Ductal	Died 2 years-without recurrence
Zagouri (2007) [30]	59	Cholecystitis	Lobular	Alive (12 months)
Manouras (2008) [45]	46	Cholecystitis		Died- 1 year
Jones (2009) [37]	84	Acute abdomen-Ruptured gallbladder	Lobular	Alive (34 months follow up).
Present report (2010)	56	Cholecystitis	Ductal	Died 5 months

All of the patients in this table are women. NM: not mentioned.

Because, breast cancer is the most common cancer in women, all the gallbladder metastases have been reported uniquely in this cohort. Compared to ductal carcinoma of the breast, lobular breast cancer is more likely to metastasize to the gastrointestinal tract [29]. Thus, the majority of cases of MBMB in our review were lobular. In a case of bilateral synchronous lobular and ductal breast, a metastatic deposit occurred in a 59 year-old woman (20 months after a mastectomy and lumpectomy) when she underwent a cholecystectomy for symptoms consistent with cholecystitis. Pathological examination of the gallbladder demonstrated lobular breast cancer [30]. In a separate case, an 81 year-old woman with a previous history of both lobular and ductal carcinoma presented with biliary symptoms ten years after the treatment of the first malignancy. She underwent a laparoscopic cholecystectomy for concerns of a malignancy [31]. Pathological examination demonstrated undifferentiated breast adenocarcinoma.

While the most common type of cancer to metastasize to the gallbladder is lobular, followed by ductal, a 62 year-old woman with a history of ductal papillary breast cancer was treated via a LC for symptomatic cholelithiasis [32]. Pathological examination demonstrated metastatic ductal papillary breast cancer.

The first case series of MBGB was publish in 2006 and included only four reported cases at the time [33]. In this review, the authors included the case of a 53 year-old woman who had undergone a modified radical mastectomy for the management of lobular carcinoma. She then developed abdominal pain and diagnostic imaging revealed the gallbladder as a potential source for which she underwent an exploratory laparotomy and a cholecystectomy. Pathological examination of the gallbladder showed lobular breast carcinoma. The authors discussed the available reports at the time. Three cases of MBGB had presented with symptoms of cholecystitis [34,35]. Cholecystitis was the most common presentation for all cases of MBGB (Table 1).

However, unusual presentations were common. Two cases of metastatic breast cancer: one to the ileum and one of the gallbladder presented symptomatically, which lead to diagnostic imaging and the unveiling of the diagnosis of MBGB [36]. An 84 year-old woman presented with an acute abdomen and free air. At laparotomy, she had a ruptured gallbladder that demonstrated to be metastatic disease originating from lobular carcinoma of the breast [37]. A similar case where a 78 year-old woman presented with bile peritonitis with the same diagnosis [38] was included in this report. In the later case, the patient died soon after exploratory laparotomy. At autopsy, she had carcinomatosis that included the sac of an incarcerated umbilical hernia [28].

In our review, lobular carcinoma is the most common type of breast cancer with metastasis to the gallbladder. Most patients in this group present with symptoms and because they were all women, a diagnosis consistent with biliary colic was entertained and a cholecystectomy performed. Most patients in this cohort had a relatively good prognosis in spite of metastatic dissemination. While there are no studies comparing the laparoscopic to open approach, a substantial number of these cases were performed laparoscopically such that it is possible to proceed with such approach in a case-to-case basis.

### Miscellaneous malignancies metastatic to the gallbladder

In a Korean review of metastatic lesions to the gallbladder, the most common site of origin for the primary cancer originated from the gastrointestinal tract with the stomach (n = 8) and colon (n = 3) as the most common sites. The authors presented two lesions metastatis to the GB from the HCC, RCC, and melanoma as well as one from the extra hepatic bile ducts, uterus, and appendix. The vast majority of these cases were metachronous lesions and symptomatic. The authors concluded that a complete resection of gross disease was associated with the best chance of survival in this cohort of patients [2]. However, because of the high incidence of gastric and hepatic cancers in the Asian population, these findings might be the reflection of such geographic specific-type malignancy prevalence. Other uncommon sites of origin metastatic to the gallbladder appear in the literature.

#### Small cell cervical cancer

A report of a 60 year-old woman with symptomatic cholelithiasis who underwent a laparoscopic cholecystectomy was previously reported. Pathological examination of the gallbladder and oncologic diagnostic workup demonstrated synchronous metastatic gallbladder cancer form a cervical primary site. She succumbed to the progressive nature of this disease 16 months after the diagnosis [39].

#### Rectal Adenocarcinoma

A case of an 83 year-old men with locally advanced adenocarcinoma of the rectum underwent perianal resection following neoadjuvant chemoradiation. Because of symptomatic cholelithiasis, he had a concomitant LC. Pathological examination of the gallbladder demonstrated metastatic rectal adenocarcinoma, which in 2008 was the first described such case and no other such cases were found in our review [40].

#### Lung cancer

A 45-year old man developed symptomatic cholecystitis form a metastatic lesion with histological origin of non-small lung cancer [41]. In a second case report, a 69 year-old man with inoperable squamous cell carcinoma of the lung developed cholecystitis from a metastatic deposit from this malignancy [42]. He underwent an open cholecystectomy with improvement of symptoms. Pathological examination confirmed the diagnosis.

## Conclusions

Metastatic melanoma is the most commonly found deposit in the gallbladder. Clear indications for surgical intervention are disease limited to the gallbladder and symptomatic disease for palliation. Laparoscopic cholecystectomy without a lymphadenectomy appears to be the most consensus agreement in the literature. Other cases for metastatic melanoma to the gallbladder must be addressed in a case-to-case basis. RCC metastatic to the gallbladder appears to have a good prognosis for cure and most of these cases necessitate a cholecystectomy, which might be approached laparoscopically. Patients with metastatic breast cancer to the gallbladder are women who typically present with symptoms and a history of lobular breast cancer. Because of symptomatic disease, a cholecystectomy is invariably the rule and this can be approached laparoscopically. Metastatic disease from other malignancies should be addressed in a case-to-case basis.

## Competing interests

The authors declare that they have no competing interests.

## Authors' contributions

ZK conceived the study, performed chart review, literature search and drafted the manuscript. JH helped with chart review and revision of the manuscript. PK provided pathology images. SH made revisions to manuscript and participated in study design and coordination. All authors read and approved the final manuscript.

## Consent

Consent for patient in the case report was obtained from the family of the deceased.
